# Memoir of the Late James Hope, M.D., Physician to St. George's Hospital

**Published:** 1842-07-01

**Authors:** 


Memoir of the late James Hope, M.D. Physician to St. George's Hospital, &c. &c. By Mrs. *Hope*. To which are added Remarks on Classical Education, by Dr. *Hope*; and Letters from a Senior to a Junior Physician, by Dr. *Burder*. The whole Edited by *Klein Grant*, M.D. &c. &c. London, Hatchard and Son, 12mo. pp. 358.

The loss which the profession and the public have experienced in the person of Dr. Hope, is too recent and too severe to have worn out of their memories. A Memoir of him will rather perpetuate than revive the recollection of his talents and his goodness, and will form a melancholy yet pleasing boon to those who knew and esteemed him.

The life of a physician is too little chequered by events of magnitude, to present those charms of adventure and vicissitude which captivate the public mind. Dr. Hope's is no exception to the rule. Yet there are some incidents and traits of character interesting, if not instructive to those who are treading in the same walk of life, and have earned or wish to earn that success, a large share of which was accorded to him.

James Hope was the descendant of a respectable Scotch family. His father was a merchant and manufacturer at Stockport, and retired from business to a library and garden, with a good fortune, at the age of forty-four. He died in 1838, at the age of eighty-five. Dr. Hope was the tenth child of a family of twelve, and was born at Stockport, on the 23rd February, 1801. Of this family of twelve, only four survived. Five died under the age of twenty-five; two others, including Dr. Hope, died at forty; and the four surviving members of the family, are of a remarkably delicate constitution. Five of the eight have died of tubercular disease, so that their constitutions had in them something radically wrong.

At the age of six or seven James Hope was placed at a day-school, where he acquired, at all events, the art of penmanship and of drawing maps, which he executed with singular beauty and correctness. There is still extant a chart of the History of England, above a yard square, done at the age of nine, and so admirably written, as well as coloured, as not to be distinguishable from an engraving. At the age of ten he went to a grammar school at Knutsford, and at twelve, to the Rev. G. S. Weidemann, who qualified boys for College. Here he read the standard authors, classical and native, and had his character, in some degree, formed by the "Rambler." To the paper which bore the title, "Life sufficient to all purposes if well employed," he ascribed the value which he placed on "fragments of time," and he used to say, that in the employment of these lay the secret of his having done so much as he had crowded into his short life. To one who knew him intimately, it is most interesting to read this paper, and to notice how literally he acted up to its precepts; and how, after the lapse of nearly thirty years, he was in the habit of addressing to his young friends admonitions exactly corresponding with those of the Rambler.

At the age of fourteen young Hope went to the Macclesfield Grammar School. Here, feeling mortified at his slight acquaintance with Greek, he proposed to a boy in the class above, to get up at four o'clock in the morning, and read through Herodotus with him. This plan, including also a portion of Thucydides, was continued for a year and a half. He had an excellent memory, and became an accomplished angler, for he not only made his own flies, but even his lines and rods, the latter being almost as neat and true as any that could be purchased. Like several other eminent men, he preserved his attachment to trout-fishing through life. He read the highest classics, shot, fished, and joined the yeomanry lancers. He became so expert in the use of the lance and broad-sword, that he was appointed fugleman to the corps, and on leaving his military calling, he was presented with a broad-sword, to which he always attached much value, and which is now in the possession of his family.

After he had spent a year at home, his father proposed to him to become a physician. To the medical profession, he had always felt the strongest dislike, and this proposition was received with corresponding dissatisfaction; but at last he consented to study physic, on condition that he should be allowed to practise in London.

In October, 1820, James Hope went to Edinburgh in order to commence his medical studies. In conformity to established usage, his first year was principally devoted to anatomy, and was to him one of disgust and unhappiness, from the extreme repugnance he felt to the pursuit. But he felt that his lot was cast, and resolved to conquer dislikes as well as difficulties. He compelled himself to the diligent and persevering study of anatomy, but he dissected in gloves and with forceps, so as never to touch the body; and so strongly rooted were his feelings, that it took two years to overcome them in any great degree, and they continued to affect him slightly even six or seven years after. He had set up for himself Dr. Baillie as his model, and saw the value of a knowledge of morbid anatomy to him who would succeed as a physician. As his biographer remarks, it is a rare thing to see a man not merely giving an ordinary share of attention to that which inspires him with disgust, but voluntarily selecting it as the subject of his peculiar study.

Dr. Hope became one of the Presidents of the Medical Society of Edinburgh, House Physician to the Edinburgh Infirmary, then House Surgeon to the Infirmary. In August, 1825, he took his degree. But he had also learned to play the flute, and he had been from boyhood a draughtsman. A copy of a small Vandervelde was thought worthy of a place in the collection of the Hon. Charles Hope, Lord President of Scotland; and a copy of Stirling Castle, by Simpson, about 3 feet by 2½, is in the possession of Professor Monro; both of whom are able connoisseurs in the art. It was principally, however, to subjects of morbid anatomy that he devoted his pencil, notwithstanding the great violence it did to his tastes and inclinations. When elected House Physician to the Edinburgh Infirmary, he began to carry into execution his idea of a work on morbid anatomy, to be embellished with plates. He employed an artist for some months, but at length discovered that it took more time to superintend and correct him, than to execute the drawings for himself. He, therefore, adopted the latter plan, and tried every expeditious mode that he could devise for curtailing the process. His general rule was, to finish each drawing at a single sitting, or, at the utmost, two, in order to avoid changes of colour in the specimen from too long exposure to the air.

In January, 1826, Dr. Hope went to London, and became a dresser at St. Bartholomew's Hospital. In the Spring of 1826, he passed his examination before the College of Surgeons, being determined to have this last proof of his competency in surgery. Immediately after this, he went to Paris. He thought he knew enough French to get on. But he found his mistake. He went to engage apartments at a private hotel, but after a pantomimic performance of some twenty minutes between himself and the landlady, it was found that neither could, in the slightest degree, understand the other; and, after laughter and reciprocal bows, he retired in despair. Having settled at another hotel, he now determined to devote twelve hours a day to the mere practice of speaking French. At the end of a month he ventured to sally forth, and having a fancy for the rooms at the private hotel, to which he had originally gone, he again waited on the landlady. On entering, he addressed her in fluent French, explained his wishes, &c. The landlady, meanwhile, with upraised arms, and an air of utter amazement, exclaimed, "Voilà, un miracle! You cannot be the same gentleman that called here a month ago, and could not speak a single word of French!" "The same notwithstanding." So he took the rooms and stopped there. He worked hard at morbid anatomy, &c. for an entire year in Paris. From La Belle France he went through Switzerland, on foot, to Italy. At Florence, a tempting proposal was made to him by Dr. Thomas. He offered Dr. Hope a gratuitous introduction to Lord Burghersh and his practice. His books shewed receipts to the amount of £1000. or £1100. per annum, and there was no opposition worth considering. This income in Florence was equal to two or three times the amount in London. Dr. Hope, however, was not to be diverted by any thing from the mark on which he had kept his eye steadily fixed, namely, London. To England, then, he returned in June, 1828, and paid a visit to his father who, on parting with him, gave him this advice :—

*First,*—Never keep a patient ill longer than you can possibly help.

*Secondly,*—Never take a fee to which you do not feel yourself to be justly entitled. And,

*Thirdly,*—Always *pray* for your patients.

A short time before his death, Dr. Hope said that these maxims had been the rule of his conduct, and that he could testify to their success.

In December, 1828, Dr. Hope passed the College of Physicians of London, as a Licentiate, and took a house in Lower Seymour-street, where he remained till his death. He seems to have thought this a mistake, for, he was *north of Oxford-street.*

Dr. Hope was now settled in London, without friends or connexions, to assist him. He was ambitious and sought professional reputation. With this view he assigned to himself the execution of the two works which he had long planned ; *viz.* A Treatise on Diseases of the Heart, and a complete work on Morbid Anatomy, illustrated by plates : and for the completion of these works he allotted seven years. To bring out the one was difficult, from the expense, to complete the other was more difficult, from the intricacy of the subject. Dr. Hope now selected St. George's Hospital, as the scene of continued study. He hoped too for a future appointment. In 1831, he was elected physician to the Marylebone Infirmary, an office worth £500. a year. Dr. Hope had now found friends —what he wanted was patients. The following anecdote may illustrate the difference between them. A gentleman, an old friend of Mrs. Hope's family, lived for several years within three doors of him, but never dreamt of trusting his life into the hands of a young man like Dr. Hope. This gentleman having been taken dangerously ill at Glasgow, was recommended by his medical adviser (Dr. Hannay, we believe) to come to town in order to consult Dr. Hope. " What," said the old gentleman, " you do not mean the man next-door to whom I have lived so many years ?"

Dr. Hope's experience led him to these conclusions—that an early marriage is far from a certain means of getting patients—that giving dinners before you can afford them is a more sure way of spending money than obtaining practice—that *dashing* is folly, if it is not ruin—and that envy pursues success.

Soon after this, Dr. Hope married, (may we add, a most amiable and accomplished lady)—he brought out his work on the heart—and resigned, in 1831, the office of physician to the Marylebone Infirmary, having been tricked by the new vestry out of the salary that had belonged to it.

In the course of the Summer of 1832, he persuaded Messrs. Whittaker and Co. to undertake the publication of the Morbid Anatomy on terms which experience had taught him to consider very advantageous. These were, that he was to provide all the drawings and lithography, and they were to be at the expence of the printing and the colouring of the plates. After having paid all their own expenses, Messrs. Whittaker agreed to divide the profits with him. After the lapse of three years Dr. Hope received between £60. and £70. for his share, a sum which would not even have remunerated him for the expence of the lithography, had he been compelled to employ a regular artist for its execution. In the Autumn of 1832, he delivered about five-and-twenty lectures at his own house, and commanded a regular attendance of from thirty to forty, which, considering that they were all practitioners, was more than he could have expected.

An observation of Dr. Holland's to Dr. Hope is a good one. At the close of the first year, Dr. Holland kindly inquired how he was getting on. Dr. Hope answered in cheerful terms, and mentioned how much he had made. "It does not signify," answered Dr. Holland, "how much or how little you have made; but what connexions have you formed, and what hold are you gaining on your patients' confidence?" Dr. Hope found the truth of this. Some families that he attended went or died off, his practice diminished, and the third year of his residence in London, that which preceded the publication of the Treatise on Diseases of the Heart, he made a smaller sum than at any other period. He then saw more than ever what must be the uncertain nature of every practice which rests solely on private connexions. From the publication of his work on Diseases of the Heart until his death, his practice steadily increased.

In November, 1834, Dr. Hope was elected assistant-physician to St. George's Hospital. Scarcely six years, says his biographer, had elapsed since Dr. Hope arrived in London with but one acquaintance, and since he had marked out for himself a path of high ambition and hard labour. He had allotted seven years for the accomplishment of that portion which depended on his own industry; but in five years and a half his work was completed, and his books were published. In a few months more he had attained the objects of his ambition.

From this time Dr. Hope relaxed a little in his habits of work. He had previously overdone it. He now restricted his labours to the ordinary working hours of mankind, going to bed at ten o'clock, and rising between seven and eight. He seldom departed from this rule during the remainder of his life, and on such occasions, eleven o'clock or midnight was the extreme limit of his vigils. Between 1832 and 1839, he collected the materials of the third edition of his Treatise on Diseases of the Heart. He used always to keep on his table a copy of the first edition, into which were bound a few blank leaves. On these, on the margins of the printed pages, and on loose scraps which he fastened in, he scribbled the most abbreviated notes of any new idea which occurred to him, and references to the cases illustrative of it. From these notes, which were written at broken intervals of time, and were scarcely perceived to occupy him even by those who lived in his house, he made such additions to the third edition that he may almost be said to have composed the work anew.

Dr. Hope's labours at St. George's, in his attendance on the out-patients, were most anxious, for their numbers were great, and his attention to them conscientious. He lectured too at the hospital on Forensic Medicine, a task undertaken for the benefit of the School, not from predilection. In the Spring of 1836 he assumed the lectureship on the practice of physic, at the Aldersgate Street School of Medicine. He was eminently successful. He held this appointment three years, and resigned it in 1839, when he was elected physician to St. George's Hospital.

We may introduce the following as an instance of the sentiment of duty and genuine benevolence which always actuated Dr. Hope. He would often spend the night in the house of a patient who was dangerously ill. These attentions were not confined to the rich. There was a gentleman of large fortune whose dying bed he had thus soothed, and whose family avowed their deep obligations to him. Grateful as they were for that kindness to which the rich are so accustomed, that they almost deem it their prerogative, they were much surprised some time after to find almost similar attentions lavished on a groom, who was seized with a dangerous complaint, requiring almost constant watching.

Up to May, 1836, Dr. Hope had never laboured under any symptoms of affection of the chest. In that month, when he had begun to find his duties at St. George's too laborious, he had a slight cough and pain in his side, which yielded immediately to a blister, and he considered himself entirely re-established. Unhappily, this was not the case. The disease which was to remove him from the scene of his professional exertions and utility had taken root. In September, 1838, Sir James Clark recommended his going abroad, but it was impossible. On the 19th of June, 1839, Dr. Chambers resigned the office of Physician to St. George's Hospital. Dr. Hope, then Assistant-Physician, was opposed by Dr. Williams, in his canvass for the office of Physician. The shock, the anxiety brought on hæmoptysis on that night, and his health broke up altogether. Dr. Hope's was not the mind to dread death, nor his the conduct to make death dreadful. He looked on it as a certainty, and prepared for it with calmness, nay, with pleasure.

"One day he met Dr. Chambers in consultation at the house of a patient, and having alluded to his approaching death, Dr. Chambers kindly answered 'that he ought not to despond, for that he would be quite well yet.' Dr. Hope stopped him, with an assurance that he needed not to be thus cheered, for that he was well aware of his condition; that, besides, the nature of Dr. Chambers' communication was not cheering, for he should be sorry to be detained long from his heavenly inheritance, and to exchange its prospect for the toils of his profession." 261.

In February, 1841, he retired from practice. On the 14th of May he died. Before his death, he was in the receipt of £4000. a year from his practice, an earnest of success of the highest order, had his life been spared.

It is impossible for any one to peruse the life of Dr. Hope without rising from the page with admiration and love for the man. Of unwearied industry, great mental powers, of the purest moral feeling, of the most sincere religious faith, an accomplished physician, an exemplary member of society, he filled his allotted station an example to other men, and, we trust, his spirit reposes with his God.

